# Comment on: ‘Development of PancRISK, a urine biomarker-based risk score for stratified screening of pancreatic cancer patients’

**DOI:** 10.1038/s41416-020-1013-5

**Published:** 2020-08-03

**Authors:** Margot De Marco, Alessandra Rosati, Maria Caterina Turco, Liberato Marzullo

**Affiliations:** Department of Medicine, Surgery and Dentistry Schola Medica Salernitana, University of Salerno, Salerno, Italy

**Keywords:** Cancer, Oncology, Tumour immunology

In their recent article on biomarker-based risk of pancreatic cancer,^1^ the authors analyse the potential value of Trefoil protein 1 (TFF1) as a component of a PancRISK panel. Our group has been long involved in studying pancreatic cancer.^2,3^ This paper particularly attracted our attention, because some members of our group have previous experience in the study of TFF1.^4,5^ In particular, we showed that copper binding, promoting the TFF1 homodimerisation, increased its motogenic activity in in vitro wound-healing assays.^4^ That finding appeared to suggest a possible activity of TFF1 on cancer cell motility.

In the same period of the publication of the article by Blyuss et al.,^1^ another article reported the anticancer activity of some copper complexes in pancreatic cancer cells.^6^ Due to the role of copper in TFF1 activity, we might speculate that the anticancer effect of copper complexes in pancreatic cancer can be due to a possible interference of the complexes with the correct TFF1–copper interaction.

We believe that these different pieces of evidence concur in supporting a role for TFF1 in pancreatic cancer, and that this topic can be worthy of further studies.

## Data Availability

Not applicable.
